# Laser-Assisted Synthesis of Cu-Al-Ni Shape Memory Alloys: Effect of Inert Gas Pressure and Ni Content

**DOI:** 10.3390/ma12050794

**Published:** 2019-03-07

**Authors:** Stefan Niedbalski, Alicia Durán, Magdalena Walczak, Jorge A. Ramos-Grez

**Affiliations:** 1 Mechanical and Metallurgical Engineering Department, Escuela de Ingeniería, Pontificia Universidad Católica de Chile, Av. Vicuña Mackenna 4860 Macul, 7820436 Santiago, Chile; sfniedba@uc.cl (S.N.); mwalczak@ing.puc.cl (M.W.); 2 Research Center for Nanotechnology and Advanced Materials (CIEN UC), Pontificia Universidad Católica de Chile, Av. Vicuña Mackenna 4860 Macul, 7820436 Santiago, Chile; 3 Departamento de Mecánica, Facultad de Ingeniería, Universidad Tecnológica Metropolitana, José Pedro Alessandri # 1242, Nuñoa, 7800002 Santiago, Chile; alicia.duran@utem.cl

**Keywords:** shape memory alloy (SMA), selective laser melting (SLM), Cu-Al-Ni alloy, martensitic transformation

## Abstract

The paper explores the applicability of laser-assisted synthesis for producing high density Cu-Al-Ni alloys with shape memory characteristics, that could be further developed towards a method of additive manufacturing of large size Cu-based shape memory alloys (SMA). The manufacturing approach consists in laser melting of elemental powder mixture in a controlled atmosphere of varying relative pressure of protective argon gas, producing alloys of 14.2 wt.% Al and Ni content varying between 2 and 4 wt.%. All the fabricated alloys are found to have attained martensitic microstructures capable of SMA specific phase transformations in the temperature range from 85 to 192 °C. Both gas pressure and content of Ni are found to affect the specific transformation temperatures, transformation enthalpies, and mechanical properties. In particular, increasing gas pressure suppresses the austenite to martensite transformation reducing microhardness. In conclusion, the selective laser melting (SLM) employed in this work is shown capable of producing high density Cu-Al-Ni SMA (porosity ≈ 2%).

## 1. Introduction

Attaining a specific shape after being permanently deformed or exposed to a change of temperature is known as shape memory effect [1], and it is a material property desired for various mechanical components in ever more applications, particularly biomedical, automotive, and aerospace fields [2]. In the case of shape memory alloys (SMA) the property is attributed to the martensitic transformation in analogy to similar process first described in steel technology. The most known and most studied SMAs are titanium and nickel alloys [3], in particular Nitinol (Ni-Ti), characterized by high stability of the martensitic phase, enabling reliable use of the shape memory effect over time. Although Nitinol has a number of other favorable properties such as mechanical strength and corrosion resistance, and has been shown suitable for laser processing [4,5] and additive manufacturing [6,7], its practical use has been restricted to high-end applications due to the elevated cost of the prime material and the necessity of employing special manufacturing techniques to contain the high reactivity of titanium. For this reason, Cu-based SMA have attracted attention not only as a low-cost alternative to the mechanical applications of Nitinol [8], but also for further applications made possible by the superior electrical and thermal conductivity, and the possibility of improving resistance to thermal aging of these alloys [9,10,11,12,13]. A further property exploited in Cu-based SMA is that of superelasticity, allowing for elastic deformations as high as 14% [14], and rendering them eligible for storage and dissipation of mechanical energy [15,16], which is particularly interesting for large-scale applications such as damping seismic vibrations in structural components [17,18]. In this work, we focus on a Cu-Al-Ni alloy due to proximity of its transformation temperatures to ambient condition, sufficient thermal stability at high temperatures [19], non-toxicity as compared with the Be-containing alternatives [20], and relatively well understood mechanism of shape memory effect, studied with detail on single crystals [21,22,23,24,25].

Although the reactivity of Cu in oxygen–rich atmosphere is not as high as that of Ti, it still poses a challenge for a fabrication of Cu-Al-Ni SMA of arbitrary shape. The strategies that have been reported successful include: melting pure elements in an argon atmosphere and quenching to obtain Cu-11.92%Al-3.78%Ni [26]; melt-spinning in vacuum furnace to fabricate Cu-Al-Ni SMA ribbons of varying Al content [9]; self-propagated high-temperature syntheses (SHS) applicable to produce Cu-14.1%Al-4%Ni single crystals [27]; spark plasma sintering of Cu-13.01%Al-3.91%Ni, microalloyed with Ti and Cr, prepared from prealloyed powder by spark-erosion in liquid argon [28]; directional solidification to produce polycrystalline Cu-13.5%Al-4%Ni [29]; hot rolling of alloy powder to produce Cu-14.1%Al-4.5%Ni [30]; and DC magnetron sputtering—a physical vapor deposition (PVD) technique—to deposit thin films of Cu-21%Al-7%Ni [31]. Recently, laser processing has also been shown effective for producing Cu-Al-Ni with SMA-specific intermetallic phases [32,33]. In particular, application of ytterbium laser of 50–70 W in a protective gas shield was shown applicable for fabricating Cu-14%Al-4%Ni from powder mixture of Cu-Ni alloy and elemental Al [34,35].

Although the laser-assisted approach has the highest potential of developing towards additive manufacturing technology [36], it involves a large number of parameters that can alter the mechanical and physical properties of the final piece [37] requiring exploration of the effect of each parameter individually and possible cross-effects with other parameters. In particular, it is convenient to understand metallurgy of the selected alloy, before moving towards fabrication of single tracks [38], not to mention shapes of higher complexity. This study focuses on the challenge posed by the affinity of Cu and Al to atmospheric oxygen for which two main strategies have been pursued so far: (i) suppressing the oxidation by including a fluxing agent to the processed powder [39,40], or (ii) processing under the protective atmosphere of a non-reactive gas [26,27,28,29,41] or in vacuum [42].

Whereas typical SLM procedure relies on recirculating an inert gas shield [43], our first trial to produce Cu-14.2%Al-3%Ni SMA with specific martensitic phase transformations employed an extensive degassing (24 h) procedure [44]. In this paper, we explore the effect of gas pressure allowed in the vacuum/controlled atmosphere chamber during the process. In addition, the effect of amount of alloyed nickel on the resulting microstructure and SMA transformation temperatures is explored.

## 2. Materials and Methods

### 2.1. Fabrication Procedure

Alloys of the nominal Ni content of 2 wt.%, 3 wt.%, and 4 wt.% were fabricated maintaining the content of Al constant at 14.2 wt.% and completing the composition with Cu. The elemental powders were purchased from Sigma-Aldrich (Santiago, Chile) with purities and particles sizes indicated in Table 1. The powders were weighed using an analytic balance to produce a total of 10 g of powder mixture. An example of mixture composition used for the Cu-14.2%Al-3%Ni alloy is also included in Table 1.

**Figure 1:**
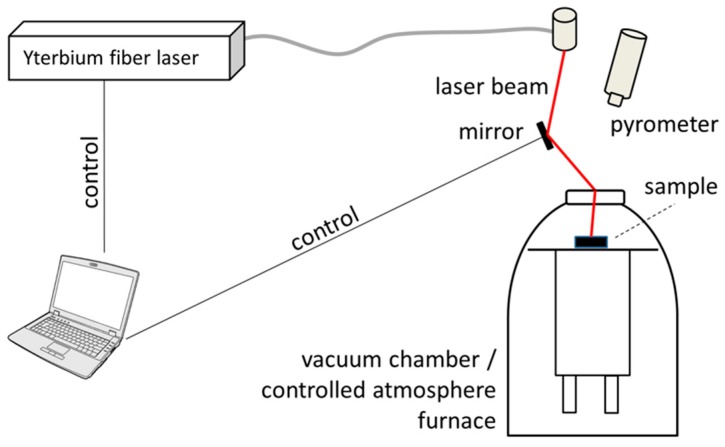
Schematic view of the experimental set-up used for the alloy fabrication.

Each powder mixture was manually macerated in 8 mL of ethanol for 10 min to produce a homogeneous paste. The paste was transferred to a rectangular tray ceramic crucible to fill its 1 cm width and 1 cm depth. The crucible was then located in a vacuum furnace (model Edwards High Vacuum Ltd. series 1200, Edwards, West Sussex, UK). The furnace was coupled with a laser set-up as shown in Figure 1, where the laser beam was reflected by a polished copper and reaches the sample to be processes through an optical window (BK7 silica glass). Immediately after the transfer, an absolute air pressure of 0.3 mbar was maintained for 30 min to assure alcohol evaporation, and then the furnace was purged with argon until the inner pressure reached 2 bar relative to atmospheric pressure. Then an ytterbium laser (IPG Photonics model YLR-300-MM-AC-Y11, IPG Photonics, Oxford, MA, USA), wavelength 1.070 mm, operated at 232 W, was fired over the powder bed at three different locations illuminating each spot for 0.3 s, and thus producing three replicas of given alloy. In the process, temperature was determined by a pyrometer (Raytek 3000 Marathon Series, Raytek Corp., Everett, WA USA) revealing 1750 °C reached at the central spot, whereas the borders of the crucible were found to reach 700 °C. Then, the inner pressure was reduced to 1 bar relative to the atmospheric pressure and then to atmospheric pressure (i.e., 0 bar relative pressure). After each pressure reduction step the laser processes were repeated directing the laser beam to a different spot by changing the angular position of the mirror.

After completing the process, air atmosphere was re-established and the solidified pieces retrieved from the crucible for analysis, separating the produced pieces from the non-processed powder.

In order to produce a martensite-rich reference, one of the specimens produced with 4% Ni at the relative pressure of 0 bar, further referred to as the HT sample, was subject to a post-processing heat treatment by austenitizing at 800 °C and quenching to ambient temperature in water with stirring.

### 2.2. Characterization

The overall appearance and inner structure of the as-fabricated samples were analyzed using micro-computed tomography (μ-CT), Skyscan 1272 by Bruker (Kontich, Belgium). The data obtained from X-ray scanning was processed using CTvox software (Version 3.3, Bruker, Kontich, Belgium) to digitally reconstruct the external shape of the pellets and to examine sections. Because of the metallic nature of the samples, only cavities and no details of the microstructure could be visualized.

For examining the inner microstructure, low speed diamond saw (IsoMet, Buehler, Lake Bluff IL, USA) was used to cut the samples. For metallographic analysis, the latter were ground using SiC paper starting from 320 down to 1200 grit, then polished using alumina paste and finally etched using a solution of iron chloride (2.5 g FeCl_3_·6H_2_O, 48 mL methanol, 10 mL HCl). The time of etching was 90 s for samples with low Al content (14.2 wt.%) and 60 s for the others. Optical imaging was performed using Nikon OPTIHOT-100 microscope (Nikon, Tokyo, Japan) and the images were digitalized by the Moticam 2300 system (Motic, Xiamen, China) for further image processing completed using ImageJ (Version 1.51, public domain Java image processing) [45]. Scanning electron microscopy (SEM) LEO 1420VP (Zeiss, Jena, Germany) equipped with energy dispersive X-ray analysis (EDX) from Oxford Instruments (INCA Energy, High Wycombe, UK) was used for imaging and elemental analyses using a 25 kV acceleration voltage. In order to determine the content of oxygen, the sample was placed in a tin capsule, melted in a furnace (LECO EF-400, LECO Corp., Saint Joseph, MI, USA), and then analyzed using the infrared cell of an oxygen analyzer (LECO TC-436DC), providing values with precision of 0.0001 wt.% for Cu. X-Ray Diffraction (XRD) analysis was employed for the identification of the crystalline phases using Siemens D5000 (Siemens, Germany) with Cu Kα1 source operated at 40 kV and 30 mA. Overview scans were collected in Bragg-Brentano configuration in the angular range of 2θ from 25° to 85°.

Temperatures and enthalpies of phase transformation were determined by differential scanning calorimetry (DSC) conducted in a nitrogen rich atmosphere using Perkin-Elmer DSC-6000 (PerkinElmer, Waltham, MA, USA) at the scanning rate of 10 °C·min^−1^. For each sample only one thermal cycle was measured. Temperature hysteresis ($\Delta T_{H}$) were calculated from the heat flow versus temperature graphs using Equation (1):

(1) $$\Delta T_{H}=\frac{1}{2}\left(A_{f}+A_{s}\right)-\frac{1}{2}\left(M_{f}+M_{s}\right),$$

where $A_{s}$, $A_{f}$, $M_{s}$, and $M_{f}$ are the transformation temperatures of austenite-start, austenite-finish, martensite-start, and martensite-finish, respectively. Transformation enthalpies of the austenite ($\Delta H_{a}$) and martensite ($\Delta H_{m}$) formations were calculated from the area under the endothermic and exothermic peaks curve in the heat flow versus temperature graphs. The procedure consisted in determining positions of the second derivative being zero corresponding to the curvature trending towards the baseline of the DSC curve. Finally, Vickers microhardness was determined using the Leco M-400-H testing machine (LECO Corp., Saint Joseph, MI, USA) with a test load of 100 g applied over 10 s.

## 3. Results

### 3.1. Chemical Composition

**Figure 2:**
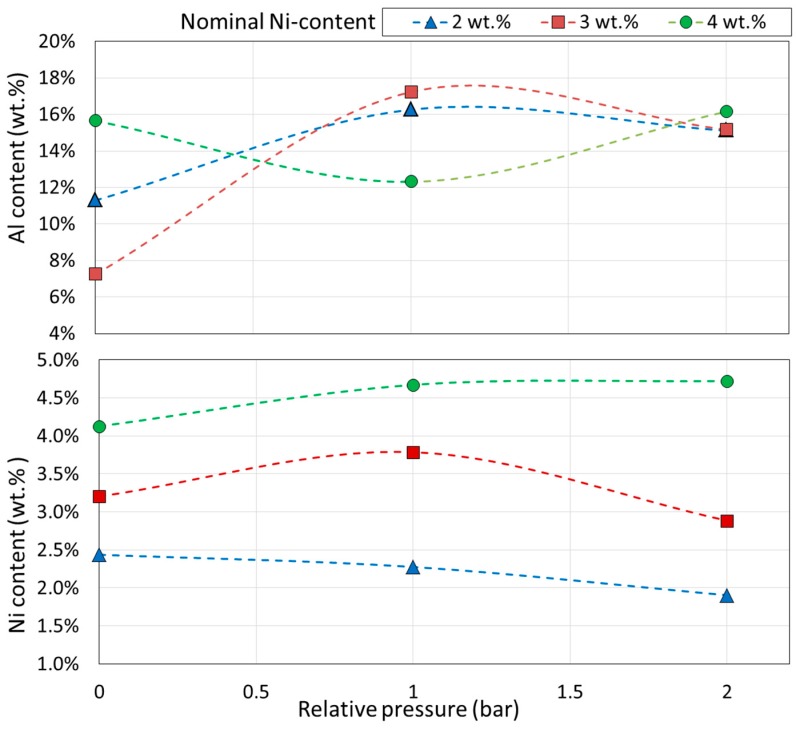
Elemental content of Al and Ni determined via EDS analysis in specimens fabricated at varying relative pressures. The dotted lines are added for visual clarity.

The contents of aluminum and nickel in the produced samples as a function of relative pressure used during laser application are shown in Figure 2. Both elements deviate from their nominal content by a consistent amount of ±0.25 wt.% in the case of Ni, whereas in the case of Al, the difference diminishes with increasing relative pressure resulting in Al content varying between 7.8–15.9 wt.%, 12.1–17.7 wt.%, and 14.6–16.0 wt.% for the relative pressures of 0, 1, and 2 bar (relative to atmospheric pressure), respectively.

**Figure 3:**
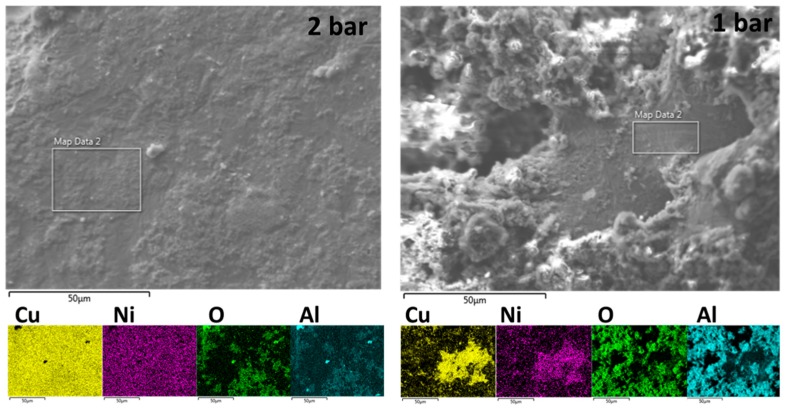
Surface appearance and elemental distribution maps of Cu, Ni, O, and Al determined using SEM-EDS on sample with nominal content of 3 wt.% Ni at relative pressures indicated in the images.

The spatial distribution of Cu, Ni, O, and Al over the surface of the produced samples is shown in Figure 3. Homogeneity of the elemental distribution was observed for copper and nickel only in samples produced in the condition of 2 bar relative pressure.

The volumetric content of oxygen in all the samples is shown in Table 2 along with porosity data determined via metallographic analysis. For samples with 2 and 3 wt.% of Ni, a general tendency of lower oxygen content for higher values of relative pressure was observed, whereas 4 wt.% produced no such tendency. In two cases (3 wt.% Ni/0 bar relative, 4 wt.% Ni/1 bar relative) oxygen content was one order of magnitude higher than the rest of the samples.

### 3.2. Microstructure

**Figure 4:**
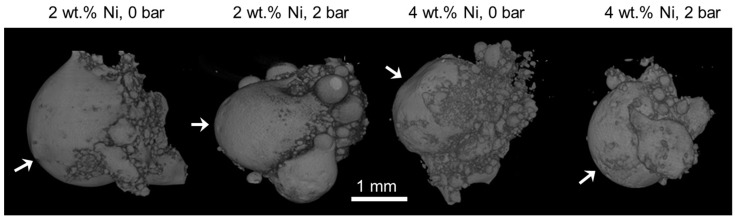
Overall shape of the produced samples reconstructured from μ-CT scans. The arrows indicate the direction of laser beam incidence during the fabrication process.

The general appearance of the produced samples, as reconstructed from the μ-CT scans, is shown in Figure 4. In all cases, the resulting pellet was about 3 mm in diameter, equidimensional in all directions, with spherical protuberances appended to the external surface. The continuous convex shape was the largest at the zone of laser beam incidence, whereas the bottom part, corresponding with the powder bed, concentrated protuberances of the smallest sizes.

**Figure 5:**
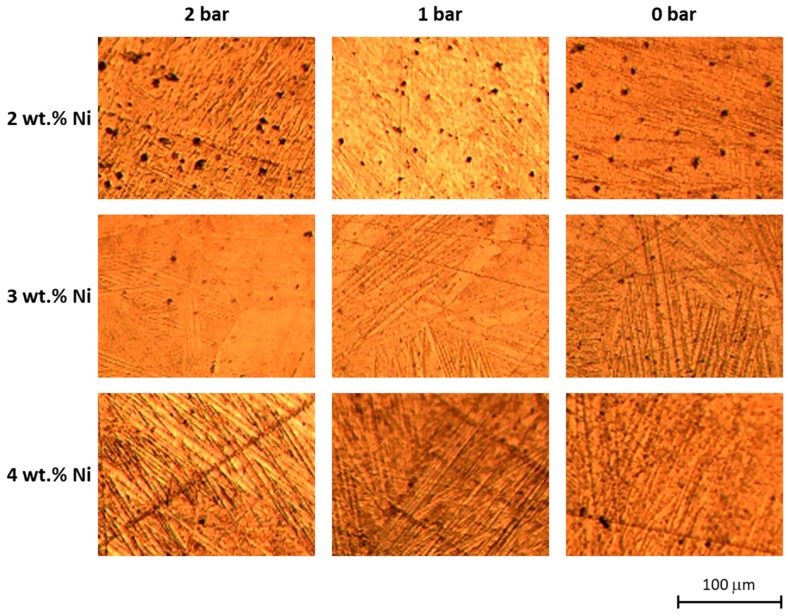
Microstructure of the alloys fabricated at the indicated conditions of relative pressure and nominal Ni content observed under optical microscopy. The scale bar is common to all micrographs.

**Figure 6:**
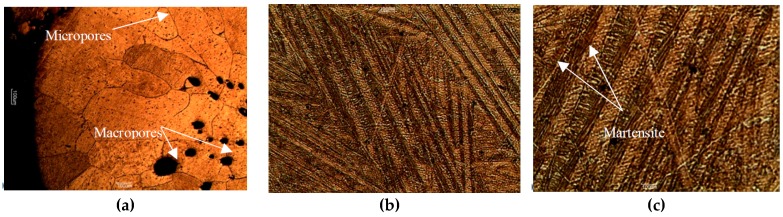
(**a**) Microstructure of alloy Cu-14.2%Al-2%Ni fabricated at the relative pressure of 2 bar and observed at 50× magnification. (**b**) Microstructure of alloy Cu-14.2%Al-3%Ni fabricated at the relative pressure of 1 bar and observed under polarized light at 500× magnification. (**c**) Same as (**b**) but observed at 1000× magnification.

**Figure 7:**
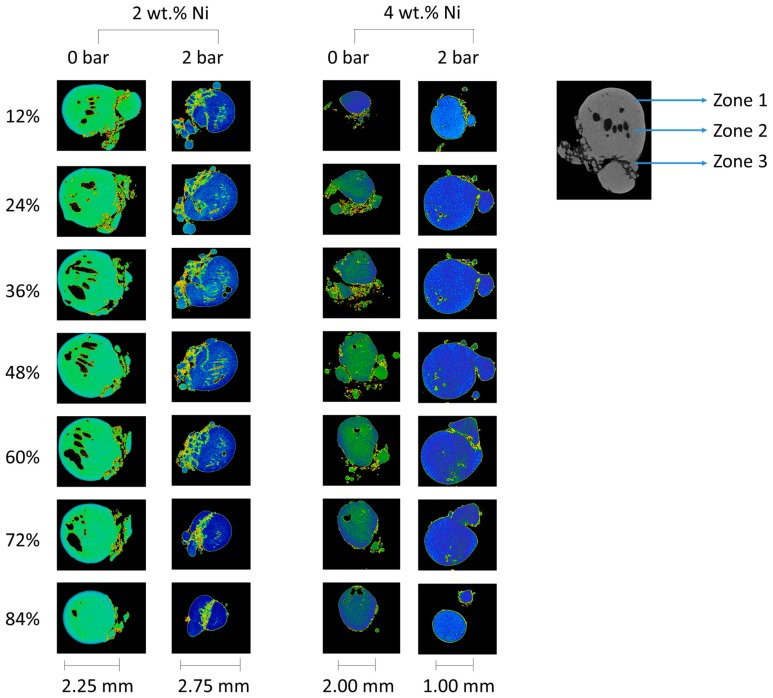
Internal structure of samples revealed by μ-CT analysis. Cross-sections are visualized in function of percentage of total length in direction perpendicular to the image. Color is used to visualize relative difference in density: blue—high, red—low. Description of division in the three zones is described in the text.

Microstructure of the bulk is presented in the metallographic images in Figure 5 and Figure 6. In some of the micrographs in Figure 5, individual remnant marks of the preparation process are visible, characterized by extending throughout the image and having no preferred orientation with respect to the microstructure. In all cases, a characteristic martensitic phase structure of “W” morphology was observed. The polycrystalline nature of the obtained alloy was also apparent, with grain sizes ranging from about 100 µm to 300 µm. In no case, star-like dendrites associated with γ_2_ phase reported in similar alloys [44,46] were observed here. Further, dark inclusions were observed, especially in samples of 2 wt.% Ni, which could be tiny and dispersed within an individual grain like those in Figure 5, or larger and spanning over two grains like those in Figure 6a. These “inclusions” might correspond with aluminum oxides but no such compound was detected and the “inclusions” were further interpreted as micro- and macroscopic pores instead. This observation corroborates with results of the μ-CT shown in Figure 7, in which dark pixels are associated with high transmission of X-rays that are expected for spaces not occupied by any component of the microstructure. The resolution of μ-CT allows for quantification of the macroscopic pores, which were found to be an average size of 50 µm. However, the possibility of the smaller pores to correspond with dislocation etch pits should not be discarded as a martensitic structure is known to be accommodated by edge dislocations at the interface with the martensite lathes. This hypothesis is supported by the submicron size of these defects apparent in Figure 5 along with their absence in the non-etched surfaces observed in SEM. Moreover, when observed under polarized light (Figure 6b,c) the 3 wt.% Ni specimen, fabricated at 1 bar relative pressure, shows the inner “W” lathe structure having widths of approximately 150 mm characteristic of the martensite and its variants. These individual lathes were spaced by approximately 20 μm, which is indicative of a high percentage of the transformed volume. Even though most of the lathes are parallel, overlapping of some lathes is also observed in Figure 6c.

In the internal structure of the pellets, three distinctive zones are identified, from bottom: (1) nearest the powder bed, characterized by a mixture of two densities, as determined by absorption of X-rays; (2) intermediate zone, between the powder bed and the zone of laser interaction, with pores of size decreasing with increasing gas pressure and nickel content; and (3) laser interaction zone, concentrating most of the porosity. The micrographies in Figure 5 correspond with zone 2.

For visualization of the mixed densities of zone 1, artificial coloring was used, employing a rainbow color scale and assigning blue to the highest X-ray absorption, i.e., highest mass density, and red to the lowest.

Shape of the internal macroscopic pores in zone 2 is revealed to be elongated along the direction of laser incidence. The best internal quality of the pellet is obtained for the highest pressure and highest Ni content, corroborating with the lowest porosity determined via metallographic analysis (Table 2).

### 3.3. XRD Analysis

**Figure 8:**
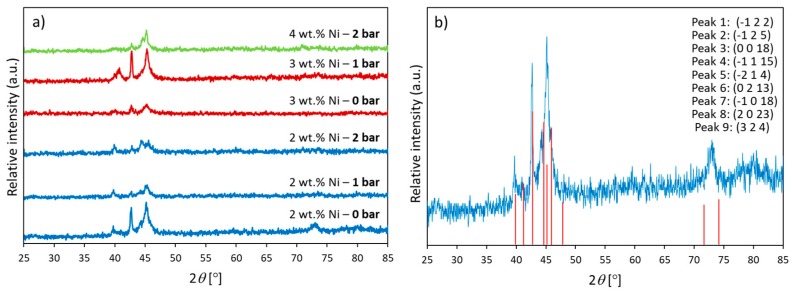
XRD analysis: (**a**) comparison of diffraction patterns from different samples, and (**b**) identification of peaks by comparing pattern of sample with 2 wt.% Ni produced at 0 bar relative pressure with peak positions and relative intensities of the reference pattern. The associated crystallographic planes are indicated in order of peak appearance from the lowest to highest diffraction angle.

X-ray diffraction patterns of the processed samples are shown in Figure 8. All patterns are of the same type with differing relative peak heights. The best match for the pattern was found to be the reference 50-1247 of the PDF2 database, corresponding with monoclinic compound of chemical formula Al_7_Cu_23_Ni. The crystallographic planes associated with each peak are indicated in Figure 8b. This compound was also identified as a continuous-casted hypoeutectic Cu-13%Al-4%Ni and associated with β′_1_-martensite [47]. The compound is, however, different from the Al_7_Cu_4_Ni intermetallic alloy found in laser-processed Cu-14%Al-4%Ni by other authors [34,35]. The quality of the diffraction peaks assessed via the full width at half maximum was the best for samples with 2 wt.% Ni 0 bar and 3 wt.% Ni 1 bar.

### 3.4. DSC Analysis

**Figure 9:**
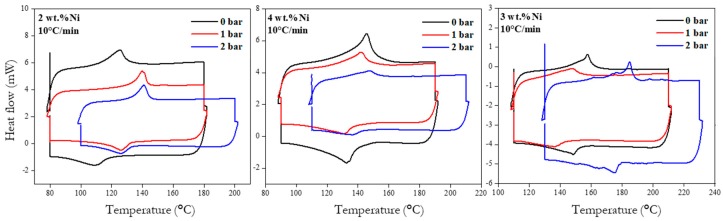
DSC curves obtained at 10 °C·min^−1^ for samples fabricated with the indicated content Ni at the relative pressure of 2 bar (blue line), 1 bar (red line), and 0 bar (black line).

The DSC curves, recorded for all the samples are shown in Figure 9, whereas the derived transformation temperatures and temperature hysteresis are summarized in Table 3. All the DSC curves show one peak in the forward (heating) direction and one peak in the backward (cooling) direction. Considering that martensite was identified in the as-synthesized materials, the peak in the heating direction is associated with martensite to austenite phase transformation with $A_{s}$ and $A_{f}$ marking the corresponding temperatures of start and finish. In the cooling direction, austenite was transformed back to martensite with respective start and finish temperatures of $M_{s}$ and $M_{f}$.

All the martensite transformation temperatures ranged from 85 to 182 °C, whereas austenite transformations were found between 98 and 192 °C. The values of $M_{s}$ and $A_{s}$ were on average lowest in samples of 2 wt.% of Ni, followed by 4 wt.% Ni, and the highest in case of 3 wt.% Ni. The effect of pressure is noted in that highest phase transformation temperatures were observed for samples processed under 2 bar relative pressure, whereas the lowest temperatures were observed for the series processed under 0 and 1 bar.

The transformations enthalpies computed from the DSC graphs as the area under the respective endothermic and exothermic peaks did not reach the value of 10 J·g^−1^ expected for full transformation in this type of alloy [12,48]. The highest enthalpy value corresponded to 8.73 J·g^−1^ and it was achieved by the quenched 4 wt.% Ni specimen (sample HT). This observation indicates that both volumetric transformations, i.e., for austenite (on heating) and martensite (on cooling), were completed only partially in all the laser processing. However, it can be observed that the austenite and martensite transformation enthalpies associated with heating and cooling, respectively, were reduced further with increasing pressure suggesting that less volumetric transformation took place with increasing atmospheric pressure. This observation suggests that pressure hindered the transformation in both directions. The effect of the wt.% of Ni did not alter enthalpies values significantly, except for the specimen with 3 wt.% Ni, which was reduced for relative pressures of 1 and 2 bar in both transformation directions. In particular, the specimen containing 2 wt.% Ni produced at 1 bar relative pressure showed a discrepancy as its computed enthalpy during heating was higher than expected when compared to the overall decreasing trend. Moreover, the computed enthalpy obtained for the specimen containing 3 wt.% Ni produced at 0 bar relative pressure, which resulted in a value close to the HT value, is explained by the lowest effective content of Al (Figure 2), consistent with the highest oxygen content (Table 2), that changed the avalanche criticality of the alloy [49,50,51], affecting the kinetics of transformations through instability and thus the volume percentage transformed in time.

The temperature hysteresis ranged from 9 to 28 °C and its specific value was more associated with the content of nickel rather than pressure in the laser processing chamber. However, it is known that martensitic transformation temperatures for this alloy varies significantly with the degree of the DO3 order, which in turn is very likely to depend on the quenching rate. The quenching rate experienced by these specimens from the superheated melt down to room temperature was estimated to be thousands of degrees per second; however, less when compared to typical self-quenching in laser processing, as the underlying material is now a powder of lower heat conductivity.

### 3.5. Microhardness

**Figure 10:**
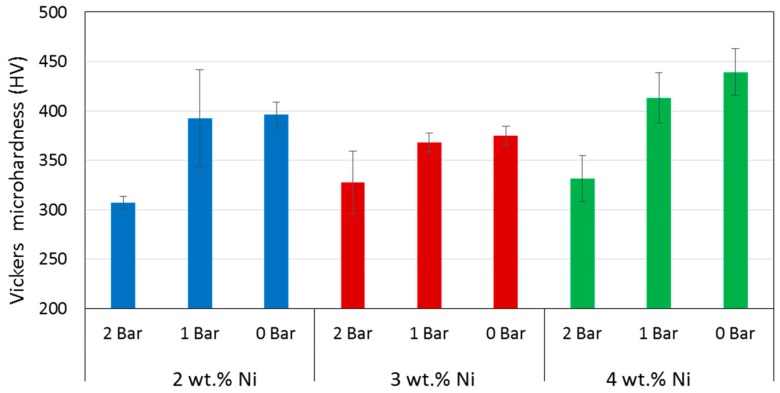
Summary of Vickers microhardness determined for the alloys.

The values of Vickers microhardness (HV) determined for all the fabricated alloys are summarized in Figure 10, in which a negative correlation with chamber pressure was observed, i.e., the higher the pressure, the lower the hardness. The overall average values for given Ni content were 356 ± 41, 357 ± 21, and 394 ± 46 HV for the 2, 3, and 4 wt.% Ni, respectively, showing no clear trend of hardness with the chemical composition.

## 4. Discussion

**Figure 11:**
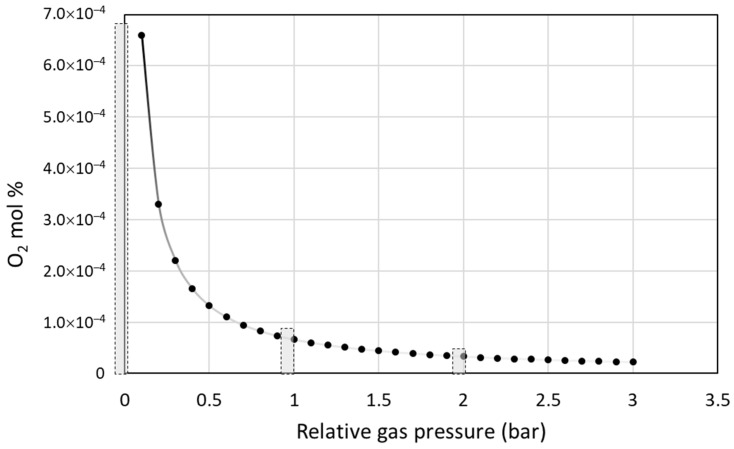
Content of residual oxygen in the processing chamber corresponding with the relative gas pressure established with Ar gas over the residue pressure of 0.3 mbar. The bars indicate conditions explored in this study.

For discussing the results presented above, the effective oxygen content in the laser processing chamber has to be considered. Although the chamber was evacuated prior to application of the laser, the residual gas of 0.3 mbar still contained air, which upon introduction of argon contributed oxygen to the gas mixture. Supposing the amount of residue air to remain constant, the molar fraction of oxygen can be calculated to change with the total gas pressure as shown in Figure 11.

The amount of oxygen determined in the fabricated pellets was generally lower for the higher pressures, with the exception of the 4 wt.% Ni sample series (Table 2). This latter inconsistency did not corroborate with the microstructural analysis and is thus considered to be result of incautious sample manipulation, interfering with the oxygen content, which happens to be particular for the case of the sample fabricated with 4 wt.% Ni at 1 bar relative pressure. Moreover, the size of the resulting pellet was also indicative of the time available during the processes contamination with oxygen. According to Chvorinov’s rule, a smaller diameter of a produced piece is associated with shorter solidification times, providing shorter effective exposition to oxygen adsorption, especially at the higher relative pressures.

Elemental composition of the obtained alloys (Figure 2) deviated from the nominal values of Al and Ni set by weighing the powders. Such discrepancies are not uncommon in laser processing and even stronger deviation was reported for laser-synthesized Cu-Al-Ni by Shishkovsky et al. [34], who explained it as occurring via formation of the intermetallic phases and deficient mixing of the involved elements, in particular the incomplete dissolution of CuNi_10_ particles of the precursor powder. In this work, elemental powders and a higher laser beam power were used. Taking into account the local temperature increase measured by the pyrometer to be above the melting point of Al, evaporation of this element should also be expected, especially at the lower total pressure [52]. This explanation is supported by the model of selective laser melting with evaporation proposed by Verhaeghe et al. [53].

The “W”-type morphology observed in all the synthesized samples (Figure 5) is characteristic of martensitic phases of Cu-based SMAs of similar chemical composition reported by other authors [22,25,27,47]. Identification of the type of martensite based on microstructure is not straightforward considering that the ranges of Al and Ni content are in the vicinity of eutectoid composition [54]. However, the presence of a martensitic microstructure is further confirmed using Figure 6b,c, which when observed under polarized light and higher magnification, clearly exhibited a microstructure of martensitic lathes and their variants. Although metallographic analyses of other authors, e.g., References [25,30,55], allowed for associating the martensite of irregular zig-zag morphology and martensite with parallel plates with the β’_3_ and γ’_3_ phases, respectively (here the subindex 3 was used as suggested by Reference [25]), it was the XRD analysis that revealed the β-derived martensite in all our samples (Figure 6). It should be noted, that no other peaks were observed in the diffraction patterns, indicating that all of the precursor powders were incorporated into the alloy and that all of the molten metal had attained martensitic microstructure, with no residual β or γ phases. The relatively low quality of peaks in terms of full width at half maximum indicated the submicron size of the crystalline martensite domains as estimated by the Sherrer’s relation between crystalline size and peak width. In further development of the technique towards additive manufacturing, the possibility of crystallographic texture to emerge will have to be taken into account as it is likely to be produced in SLM [56,57,58,59] and has been shown crucial to fatigue resistance of other SMAs, e.g., Nitinol [60]. The small extension of martensite plates is typical of rapidly solidified polycrystalline Cu-Al-Ni SMAs as compared with single crystals [61], which in our case can be explained by the higher curvature of pellets obtained at the higher pressure, which provide conditions for faster cooling according to Chvorinov’s rule.

A further feature of the spherical specimens is their porosity, revealed by analysis of the μ-CT scans (Figure 7), in which three zones have been characterized. The zone nearest the powder bed, associated with incomplete powder mixing, was also the farthest from laser incidence attaining the lowest temperatures. Considering the temperature gradients established during and after the laser action, it can be expected that all the metal powders melted at the same time, considering their melting points of 1084, 659, and 1452 °C for Cu, Al, and Ni, respectively [52]. Extension of this zone is affected by the gas pressure, which at higher values results in a more homogenous density distribution. The higher pressure also promotes radial compaction, enhancing the heat conductivity from the laser beam into the powder bed. The second zone, intermediate between powder bed and laser interaction, is characterized by a bubble-like porosity. Size and shape of the pores depend both on the gas pressure and nickel content, attaining smaller sizes at higher pressure and nickel content. However, most of the porosity was concentrated in the last zone of laser-metal interaction. The dependence of pores’ morphology on nickel content and gas pressure can be explained by the dependence of surface tension on these parameters. Higher surface tensions should correlate with the formation of smaller pores according to Young-Laplace’s relationship, but there is no data available in literature to verify this hypothesis for this particular alloy. Another factor that should be taken into account in the formation of the three zones is the presence of admixed and adsorbed gases. These gases are released upon a rapid rise of temperature and metal melting associated with the laser action, but their transport to the surface can be obstructed by the surrounding metal being solidified. This phenomenon may also hinder propagation of heat into the melt and contribute to modification of surface tension. Higher gas pressure in the processing chamber could in principle compress the molten alloy prior solidification contributing to gas evacuation from the melt, but the effect was apparent in the shape of the specimens. Additionally, higher gas pressure resulted in specimens of smaller radii and thus underwent solidification in shorter times.

The evolution of porosity is crucial for the process of densification, which in turn, determines the mechanical performance and phase transformation characteristics of the laser-processed alloys as compared with their bulk equivalents. Insufficient densification can also compromise corrosion resistance as shown by formation of highly localized voluminous oxides in direct metal laser sintered Ti_6_Al_4_V [62]. The average porosities obtained in this study were more affected by Ni content rather than gas pressure, being 5.58, 2.83, and 1.94% for the 2, 3, and 4 wt.% Ni, respectively. These values are much lower than the 30% determined from the density data reported for Cu-Al-Ni monolayers fabricated using SLM [34], and yet could be improved considering a porosity of <0.05 vol.% obtained in Ti_6_Al_4_V alloy by tuning the processing parameters of SLM [63].

However, the most interesting property of the alloys studied here is their capacity of shape memory effect, which has been corroborated using the DSC analysis (Figure 7). All the transformation temperatures found in the synthesized alloys were higher than those reported for single crystal Cu-14%Al-4%Ni [64] and lower that those reported for Cu-14.2%Al-3%Ni obtained using direct laser fabrication [44]. Although transformation temperatures are known to be sensitive to the actual chemical composition of the Cu-Al-Ni system [65], none of the transformation temperatures fit the values that should follow from the dependence on Al and Ni content determined by Recarte et al. [25]. This deviation of transformation temperatures can be caused by one or a combination of the following effects: (i) incidence of porosity, which has been shown to broaden the DSC peaks and shift their position in the temperature axis as discussed for the SLM-fabricated Cu-14%Al-4%Ni [34]; (ii) contribution of grain boundaries to the transformation processes as discussed in case of laser surface remelting of Cu-Al-Ni-Mn [66] and various Cu-Al-Ni SMAs obtained by conventional methods [61]; (iii) temperature gradient specific to laser treatment as might be inferred from the effect of different thermal treatment on Cu-11%Al-3.38%Ni [48] and Cu-11.5%Al-4%Ni-0.25Fe [67]; and (iv) purity of the precursor as even small amounts of additional elements, such as for instance Fe possibly contaminating Al powder or Sn contaminating Cu, may have a considerable effect on the transformation temperatures [67,68,69]. Further, gas pressure in the processing chamber has to be taken into account, although its effect was indirect through all the mechanisms that relate pressure and molar content of oxygen with the microstructure, it also had a direct effect in reducing the transformation enthalpy of all specimens as its relative value increased. This suggests that the volumetric martensite transformation was hindered as pressure increased. The relatively low hysteresis of transformation is typical to this type of complex microstructures producing multiple conditions of metastability due to interplay of transformation with disorder [1]. The associated avalanche critically implies that for practical applications, the system will require external stress for stabilizing reversibility of the transformations.

Utility of superelastic SMAs is provided by their mechanical properties, which in terms of microhardness, was found equal to that of Cu-14.2%Al-3%Ni obtained by direct laser fabrication in oxygen-free atmosphere [44] for the sample processed at 0 bar relative pressure. However, the effect of pressure on microhardness is considerable, resulting in slight and considerable reduction in hardness when the relative pressure is increased to 1 and 2 bar, respectively (Figure 10). This systematic reduction of mechanical resistance with increasing pressure certainly suggests that the amount of volume transformed into martensite could have been decreased with pressure as well. This observation is consistent with the transformation enthalpies that also decrease with increasing pressure. What is more, the effect of pressure was stronger than that of microstructure as increased hardness could be expected in function of finer martensite plates determined by XRD (Figure 6), which is normally associated with higher hardness as shown for melt-spun Cu-13.2%Al-5.1%Ni ribbons [70]. Moreover, the fact that the specimens made under higher relative pressure underwent faster solidification could explain a lower oxygen contamination, which in turn rendered a purer alloy and therefore of lower resulting hardness.

## 5. Conclusions

This work explored applicability of localized laser melting for fabrication of high-density Cu-Al-Ni alloy with shape memory characteristics using a variable amount of the protective argon gas. The method is concluded to be feasible by demonstrating its capability of efficiently transforming elemental powders into SMA-specific martensite. However, a considerable relationship between the gas pressure used during laser application and the resulting porosity, hardness, phase transformation temperatures and enthalpies, and martensite’s crystallite size have been found, in particular:

(1) Higher gas pressure in the processing chamber resulted in decreased transformation enthalpies (in both directions), as well as in lower microhardness. This suggest that the principal effect for microhardness reduction is the hindering of martensite volumetric transformation as the transformation enthalpy is lowered by increasing pressure.
(2) Lower gas pressures were associated with higher molar fraction of residual oxygen, which increased the probability of oxidation, especially at short laser interaction times.
(3) Higher gas pressures were associated with shorter solidification times, diminishing the probability of oxidation and resulting in purer alloy of lower hardness.
(4) The effect of gas pressure on transformation temperatures was indirect, through microstructure, rather than direct, through enthalpy of the volume. However, the effect of gas pressure on transformation enthalpy was direct and associated with the hindering of the volumetric transformation of austenite into martensite upon cooling.
(5) Porosity of the specimens was associated with release of adsorbed gases rather than residual voids known from sintering and incomplete melting of powders.

In conclusion, metallurgical understating of the ternary Cu-Al-Ni system, especially its morphological evolution at varied gas pressures, is still required in order to develop the technique further towards a method of additive manufacturing of Cu-based SMAs.

## Figures and Tables

**Table 1:** Quality of the elemental powders used for alloy fabrication and an example of determining the quantities necessary for fabricating alloy of nominal composition Cu-14.2%Al-3%Ni.

	Element	Cu	Al	Ni	Total
Elemental powders	Average particle size (µm)	45	149	5	-
	Powder purity (wt.%)	99.7	93	99.99	-
Alloy Cu-14.2%Al-3%Ni	Alloy composition (wt.%)	82.8	14.2	3	100
	Equivalent mass (g)	8.28	1.42	0.3	10
	Real mass (g)	8.3049	1.5269	0.3000	10.1318

**Table 2:** Oxygen content in the fabricated samples determined using a LECO analyzer and porosities determined using metallographic analysis.

Nominal Content of Ni	Relative Pressure (*p* − *p*_atm_)	Oxygen Content	Porosity
2 wt.%	0 bar	0.04020 wt.%	9.33%
	1 bar	0.03704 wt.%	3.18%
	2 bar	0.03336 wt.%	4.23%
3 wt.%	0 bar	0.52080 wt.%	4.48%
	1 bar	0.02026 wt.%	1.40%
	2 bar	0.00522 wt.%	2.60%
4 wt.%	0 bar	0.00690 wt.%	2.33%
	1 bar	0.12320 wt.%	2.73%
	2 bar	0.03244 wt.%	0.76%

**Table 3:** Summary of transformation temperatures, enthalpies, and temperature hysteresis determined from the DCS analysis of all the samples.

Sample	*p* − *p*_atm_	Austenitic Transition			Martensitic Transition			Hysteresis
		*A*_s_ (°C)	*A*_f_ (°C)	Δ*H*_a_ (J/g)	*M*_s_ (°C)	*M*_f_ (°C)	Δ*H*_m_ (J/g)	Δ*T*_H_ (°C)
2 wt.% Ni	0 bar	98.30	149.63	6.84	125.37	85.38	4.46	18.59
	1 bar	118.49	150.32	5.98	136.34	98.67	6.19	16.91
	2 bar	128.69	159.36	4.41	147.16	85.66	4.20	27.62
3 wt.% Ni	0 bar	145.98	166.98	8.29	158.02	130.70	6.89	12.12
	1 bar	123.83	165.67	4.57	158.50	112.37	3.70	9.32
	2 bar	166.26	192.23	3.89	182.36	152.36	3.35	11.89
4 wt.% Ni	0 bar	115.65	161.66	6.32	151.02	96.07	6.19	15.11
	1 bar	114.85	161.34	5.52	153.83	93.00	5.49	14.68
	2 bar	124.34	167.50	4.57	158.17	112.37	3.53	10.65
4 wt.% Ni	HT	98.62	161.65	8.73	148.33	92.36	5.04	9.79

## References

[1] Otsuka K., Wayman C.M. (1999). Shape Memory Materials.

[2] Mohd Jani J., Leary M., Subic A., Gibson M.A. (2014). A review of shape memory alloy research, applications and opportunities. Mater. Des..

[3] Van Humbeeck J. (2001). Shape Memory Alloys: A Material and a Technology. Adv. Eng. Mater..

[4] Khoo Z.X., Liu Y., An J., Chua C.K., Shen Y.F., Kuo C.N. (2018). A review of selective laser melted NiTi shape memory alloy. Materials.

[5] Mehrpouya M., Gisario A., Elahinia M. (2018). Laser welding of NiTi shape memory alloy: A review. J. Manuf. Process..

[6] Elahinia M., Shayesteh Moghaddam N., Taheri Andani M., Amerinatanzi A., Bimber B.A., Hamilton R.F. (2016). Fabrication of NiTi through additive manufacturing: A review. Prog. Mater. Sci..

[7] Wang X., Kustov S., Van Humbeeck J. (2018). A short review on the microstructure, transformation behavior and functional properties of NiTi shape memory alloys fabricated by selective laser melting. Materials.

[8] Dasgupta R. (2014). A look into Cu-based shape memory alloys: Present scenario and future prospects. J. Mater. Res..

[9] Lojen G., Anzel I., Kneissl A., Krizman A., Unterweger E., Kosec B., Bizjak M. (2005). Microstructure of rapidly solidified Cu-Al-Ni shape memory alloy ribbons. J. Mater. Process. Technol..

[10] Mazzer E.M., Kiminami C.S., Bolfarini C., Cava R.D., Botta W.J., Gargarella P. (2015). Thermodynamic analysis of the effect of annealing on the thermal stability of a Cu-Al-Ni-Mn shape memory alloy. Thermochim. Acta.

[11] Balo S.N., Sel N. (2012). Effects of thermal aging on transformation temperatures and some physical parameters of Cu-13.5 wt. %Al–4 wt. %Ni shape memory alloy. Thermochim. Acta.

[12] Pereira E.C., Matlakhova L.A., Matlakhov A.N., de Araújo C.J., Shigue C.Y., Monteiro S.N. (2016). Reversible martensite transformations in thermal cycled polycrystalline Cu-13.7%Al–4.0%Ni alloy. J. Alloys Compd..

[13] Sari U., Kirindi T. (2008). Effects of deformation on microstructure and mechanical properties of a Cu-Al-Ni shape memory alloy. Mater. Charact..

[14] Qiu C.X., Zhu S. (2014). Characterization of cyclic properties of superelastic monocrystalline Cu-Al-Be SMA wires for seismic applications. Constr. Build. Mater..

[15] Liu J.-L., Huang H.-Y., Xie J.-X. (2015). Superelastic anisotropy characteristics of columnar-grained Cu-Al-Mn shape memory alloys and its potential applications. Mater. Des..

[16] Jiao Y.Q., Wen Y.H., Li N., He J.Q., Teng J. (2010). Effect of solution treatment on damping capacity and shape memory effect of a CuAlMn alloy. J. Alloys Compd..

[17] Chang W.-S., Araki Y. (2016). Use of shape-memory alloys in construction: A critical review. Proc. Inst. Civ. Eng. Civ. Eng..

[18] Ozbulut O.E., Hurlebaus S., Desroches R. (2011). Seismic Response Control Using Shape Memory Alloys: A Review. J. Intell. Mater. Syst. Struct..

[19] Miyazaki S., Otsuka K. (1989). Development of Shape Memory Alloys. ISIJ Int..

[20] Edmunds W.M. (2011). Encyclopedia of Environmental Health.

[21] Picornell C., L’vov V.A., Pons J., Cesari E. (2006). Experimental and theoretical study of mechanical stabilization of martensite in Cu-Al-Ni single crystals. Mater. Sci. Eng. A.

[22] Xiangyang Z., Qingping S., Shouwen Y. (2000). Non-invariant plane model for the interface in CuAlNi single crystal shape memory alloys. J. Mech. Phys. Solids.

[23] Sakamoto H., Shimizu K., Otsuka K. (1985). A detailed observation on successive stress-induced martensitic transformations in Cu-Al-Ni alloy single crystals above Af. Trans. Jpn. Inst. Met..

[24] Otsuka K., Saxena A., Deng J., Ren X. (2011). Mechanism of the shape memory effect in martensitic alloys: An assessment. Philos. Mag..

[25] Recarte V., Pérez-Sáez R.B., Bocanegra E.H., Nó M.L., San Juan J. (2002). Influence of Al and Ni concentration on the martensitic transformation in Cu-Al-Ni shape-memory alloys. Metall. Mater. Trans. A Phys. Metall. Mater. Sci..

[26] Sarı U., Aksoy İ. (2008). Micro-structural analysis of self-accommodating martensites in Cu–11.92wt%Al–3.78wt%Ni shape memory alloy. J. Mater. Process. Technol..

[27] Suresh N., Ramamurty U. (2008). Aging response and its effect on the functional properties of Cu-Al-Ni shape memory alloys. J. Alloys Compd..

[28] Portier R.A., Ochin P., Pasko A., Monastyrsky G.E., Gilchuk A.V., Kolomytsev V.I., Koval Y.N. (2013). Spark plasma sintering of Cu-Al-Ni shape memory alloy. J. Alloys Compd..

[29] Yuan B., Zheng P., Gao Y., Zhu M., Dunand D.C. (2015). Effect of directional solidification and porosity upon the superelasticity of Cu-Al-Ni shape-memory alloys. Mater. Des..

[30] Vajpai S.K., Dube R.K., Sangal S. (2013). Application of rapid solidification powder metallurgy processing to prepare Cu-Al-Ni high temperature shape memory alloy strips with high strength and high ductility. Mater. Sci. Eng. A.

[31] Morán M.J., Condó A.M., Soldera F., Sirena M., Haberkorn N. (2016). Martensitic transformation in freestanding and supported Cu-Al-Ni thin films obtained at low deposition temperatures. Mater. Lett..

[32] Liu Y.H., Guo Z.X., Shen P., Wang H.Y., Hu J.D. (2007). Study on densification of laser ignited reaction sintering of Ni-Al-Cu powder. Sci. Sin..

[33] Yue T.M., Li T., Lin X. (2010). Microstructure and phase evolution in laser cladding of Ni/Cu/Al multilayer on magnesium substrates. Metall. Mater. Trans. A.

[34] Shishkovsky I., Yadroitsev I., Morozov Y. (2016). Laser-assisted synthesis in Cu-Al-Ni system and some of its properties. J. Alloys Compd..

[35] Volyanski I., Shishkovsky I.V., Yadroitsev I., Shcherbakov V.I., Morozov Y.G. (2016). Layer-by-layer laser synthesis of Cu-Al-Ni intermetallic compounds and shape memory effect. Inorg. Mater..

[36] Yap C.Y., Chua C.K., Dong Z.L., Liu Z.H., Zhang D.Q., Loh L.E., Sing S.L. (2015). Review of selective laser melting: Materials and applications. Appl. Phys. Rev..

[37] Spears T.G., Gold S.A. (2016). In-process sensing in selective laser melting (SLM) additive manufacturing. Integr. Mater. Manuf. Innov..

[38] Yadroitsev I., Gusarov A., Yadroitsava I., Smurov I. (2010). Single track formation in selective laser melting of metal powders. J. Mater. Process. Technol..

[39] Zhu H.H., Lu L., Fuh J.Y.H. (2003). Development and characterisation of direct laser sintering Cu-based metal powder. J. Mater. Process. Technol..

[40] Gu D., Shen Y. (2006). Development and characterisation of direct laser sintering Cu-based metal powder. Powder Metall..

[41] Cava R.D., Bolfarini C., Kiminami C.S., Mazzer E.M., Botta Filho W.J., Gargarella P., Eckert J. (2015). Spray forming of Cu-11.85Al-3.2Ni-3Mn (wt%) shape memory alloy. J. Alloys Compd..

[42] Zhang X., Sui J., Liu Q., Cai W. (2016). Effects of Gd addition on the microstructure, mechanical properties and shape memory effect of polycrystalline Cu-Al-Ni shape memory alloy. Mater. Lett..

[43] Ladewig A., Schlick G., Fisser M., Schulze V., Glatzel U. (2016). Influence of the shielding gas flow on the removal of process by-products in the selective laser melting process. Addit. Manuf..

[44] Reyes-Donoso G., Walczak M., Ramos-Moore E., Ramos-Grez J. (2017). Towards direct metal laser fabrication of Cu–based shape memory alloys. Rapid. Prototyp. J..

[45] Abràmoff M.D., Magalhães P.J., Ram S.J. (2004). Image processing with imageJ. Biophotonics Int..

[46] Wang Z., Liu X.F., Xie J.X. (2011). Effects of solidification parameters on microstructure and mechanical properties of continuous columnar-grained Cu-Al-Ni alloy. Prog. Nat. Sci. Mater. Int..

[47] Lojen G., Gojić M., Anžel I. (2013). Continuously cast Cu-Al-Ni shape memory alloy—Properties in as-cast condition. J. Alloys Compd..

[48] Dagdelen F., Gokhan T., Aydogdu A., Aydogdu Y., Adigu O. (2003). Effects of thermal treatments on transformation behaviour in shape memory Cu-Al-Ni alloys. Mater. Lett..

[49] Vives E., Ortín J., Mañosa L., Ráfols I., Pérez-Magrané R., Planes A. (1987). Distributions of Avalanches in Martensitic Transformations. Phys. Rev. Lett..

[50] Faran E., Seiner H., Landa M., Shilo D. (2015). The effects of microstructure on crackling noise during martensitic transformation in Cu-Al-Ni. Appl. Phys. Lett..

[51] Vives E., Baró J., Gallardo M.C., Martín-Olalla J.M., Romero F.J., Driver S.L., Carpenter M.A., Salje E.K.H., Stipcich M., Romero R. (2016). Avalanche criticalities and elastic and calorimetric anomalies of the transition from cubic Cu-Al-Ni to a mixture of 18R and 2H structures. Phys. Rev. B Condens. Matter Mater. Phys..

[52] Das S. (2003). Physical Aspects of Process Control in Selective Laser Sintering of Metals. Adv. Eng. Mater..

[53] Verhaeghe F., Craeghs T., Heulens J., Pandelaers L. (2009). A pragmatic model for selective laser melting with evaporation. Acta Mater..

[54] Prince A., Kumar K.C.H. (1991). Al-Cu-Ni (Aluminium-Copper-Nickel). Landolt-Börnstein Series IV/11A2.

[55] Straumal B.B., Kilmametov A.R., López G.A., López-Ferreño I., Nó M.L., San Juan J., Hahn H., Baretzky B. (2017). High-pressure torsion driven phase transformations in Cu-Al-Ni shape memory alloys. Acta Mater..

[56] Kunze K., Etter T., Grässlin J., Shklover V. (2014). Texture, anisotropy in microstructure and mechanical properties of IN738LC alloy processed by selective laser melting (SLM). Mater. Sci. Eng. A.

[57] Thijs L., Kempen K., Kruth J.P., Van Humbeeck J. (2013). Fine-structured aluminium products with controllable texture by selective laser melting of pre-alloyed AlSi10Mg powder. Acta Mater..

[58] Zhou X., Li K., Zhang D., Liu X., Ma J., Liu W., Shen Z. (2015). Textures formed in a CoCrMo alloy by selective laser melting. J. Alloys Compd..

[59] Yadroitsev I., Krakhmalev P., Yadroitsava I., Johansson S., Smurov I. (2013). Energy input effect on morphology and microstructure of selective laser melting single track from metallic powder. J. Mater. Process. Technol..

[60] Weafer F.M., Bruzzi M.S. (2016). Micromechanical investigation into the effect of texture on the fatigue behaviour of superelastic nitinol. Int. J. Fatigue.

[61] La Roca P., Isola L., Vermaut P., Malarría J. (2015). β-grain size Effects on the 18R-martensite Microstructure in Cu-based SMA. Procedia Mater. Sci..

[62] De Damborenea J.J., Arenas M.A., Larosa M.A., Jardini A.L., de Carvalho Zavaglia C.A., Conde A. (2017). Corrosion of Ti6Al4V pins produced by direct metal laser sintering. Appl. Surf. Sci..

[63] Kasperovich G., Haubrich J., Gussone J., Requena G. (2016). Correlation between porosity and processing parameters in TiAl6V4 produced by selective laser melting. Mater. Des..

[64] Recarte V., Pérez-Sáez R.B., Bocanegra E.H., Nó M.L.L., San Juan J. (1999). Dependence of the martensitic transformation characteristics on concentration in Cu-Al-Ni shape memory alloys. Mater. Sci. Eng. A.

[65] Karagoz Z., Canbay C.A. (2013). Relationship between transformation temperatures and alloying elements in Cu-Al-Ni shape memory alloys. J. Therm. Anal. Calorim..

[66] da Silva M.R., Gargarella P., Gustmann T., Botta Filho W.J., Kiminami C.S., Eckert J., Pauly S., Bolfarini C. (2016). Laser surface remelting of a Cu-Al-Ni-Mn shape memory alloy. Mater. Sci. Eng. A.

[67] Saud S.N., Hamzah E., Abubakar T., Farahany S. (2014). Structure-property relationship of Cu-Al-Ni-Fe shape memory alloys in different quenching media. J. Mater. Eng. Perform..

[68] Zhang X., Liu Q.-S. (2016). Influence of Alloying Element Addition on Cu-Al-Ni High-Temperature Shape Memory Alloy without Second Phase Formation. Acta Metall. Sin. (Engl. Lett.).

[69] Saud S.N., Hamzah E., Abubakar T., Bakhsheshi-Rad H.R., Mohammed M.N. (2016). Influence of Tin Additions on the Phase-Transformation Characteristics of Mechanical Alloyed Cu-Al-Ni Shape-Memory Alloy. Metall. Mater. Trans. A Phys. Metall. Mater. Sci..

[70] Izadinia M., Dehghani K. (2012). Microstructural evolution and mechanical properties of nanostructured Cu-Al-Ni shape memory alloys. Int. J. Miner. Metall. Mater..

